# Origins of Alterations to *Rankl* Null Mutant Mouse Dental Root Development

**DOI:** 10.3390/ijms21062201

**Published:** 2020-03-23

**Authors:** Andrea Gama, Jorge William Vargas-Franco, Diana Carolina Sánchez Mesa, Elizabeth Restrepo Bedoya, Jérome Amiaud, Sylvie Babajko, Ariane Berdal, Ana Carolina Acevedo, Dominique Heymann, Frédéric Lézot, Beatriz Castaneda

**Affiliations:** 1 Centre de Recherche des Cordeliers, INSERM UMR-1138, Sorbonne Université, Université de Paris, Laboratoire de Physiopathologie Orale Moléculaire, F-75006 Paris, France; dea.gama10@gmail.com (A.G.); carosanchez@gmail.com (D.C.S.M.); elirebe@gmail.com (E.R.B.); sylvie.babajko@crc.jussieu.fr (S.B.); ariane.berdal@univ-paris-diderot.fr (A.B.); 2 Odontologic Center of District Federal Military Police, Brasília 70297-400, Brazil; 3 Laboratory of Oral Histopathology, Health Sciences Faculty, University of Brasília, Brasília 70910-900, Brazil; acevpoppe@gmail.com; 4 INSERM, UMR-1238, Equipe 1, Faculté de Médecine, Université de Nantes, F-44035 Nantes, France; jorge.vargas@udea.edu.co (J.W.V.-F.); jerome.amiaud@univ-nantes.fr (J.A.); frederic.lezot@univ-nantes.fr (F.L.); 5 Department of Basic Studies, Faculty of Odontology, University of Antioquia, Medellin A.A1226, Colombia; 6 Department of Orthodontics, Faculty of Odontology, University of Antioquia, Medellin A.A1226, Colombia; 7 Oral Center for Inherited Diseases, Hospital of University of Brasilia, University of Brasília, Brasília 70910-900, Brazil; 8 INSERM, LEA Sarcoma Research Unit, University of Sheffield, Department of Oncology and Human Metabolism, Medical School, Sheffield S10 2RX, UK; dominique.heymann@univ-nantes.fr; 9 INSERM, UMR-1232, LabCT, CRCNA, Université de Nantes, Université d’Angers, Institut de Cancérologie de l’Ouest, site René Gauducheau, F-44805 Saint-Herblain, France; 10 Service d’Odontologie-Stomatologie, Hôpital Pitié-Salpêtrière, AP-HP, F-75013 Paris, France

**Keywords:** tooth, RANKL/RANK signaling, bone resorption, Hertwig’s epithelial root sheath, root formation

## Abstract

The purpose of the present study was to assess the early stages of development of mouse first molar roots in the osteopetrotic context of RANKL invalidation in order to demonstrate that the radicular phenotype observed resulted not only from defective osteoclasts, but also from loss of cell-to-cell communication among dental, periodontium and alveolar bone cells involving RANKL signaling. Two experimental models were used in this study: *Rankl* mutants with permanent RANKL invalidation, and C57BL/6J mice injected during the first postnatal week with a RANKL neutralizing antibody corresponding to a transient RANKL invalidation. The dento-alveolar complex was systematically analyzed using micro-CT, and histological and immunohistochemical approaches. These experiments showed that the root elongation alterations observed in the *Rankl*^-/-^ mice were associated with reduced proliferation of the RANK-expressing HERS cells with a significant decrease in proliferating cell nuclear antigen (PCNA) expression and a significant increase in P21 expression. The phenotypic comparison of the adult first molar root at 35 days between permanent and transitory invalidations of RANKL made it possible to demonstrate that alterations in dental root development have at least two origins, one intrinsic and linked to proliferation/differentiation perturbations in dental-root-forming cells, the other extrinsic and corresponding to disturbances of bone cell differentiation/function.

## 1. Introduction

Completion of proper development of the complex formed by the tooth, the periodontium and the alveolar bone has been shown to be dependent on reiterative temporo–spatial molecular signals [1,2]. The development of teeth and alveolar bone is mutually dependent in the course of development, as widely described in alveolar ridge hypotrophy, caused by tooth agenesis, and in sclerotic bone disorders such as osteopetrosis [3,4]. In the latter case, alterations in dental root development were initially considered secondary to a mechanical issue, because the roots cannot be extended within a limited space. Recent studies have suggested that, beyond this problem of space, root development is directly dependent on the bone modeling/remodeling activity level [5,6,7,8]. More specifically, it has been demonstrated that the activity of osteoclasts, controlled by the RANKL/RANK (Tumor Necrosis Factor Superfamily Member 11/Tumor Necrosis Factor Receptor Superfamily Member 11A) signaling pathway has an effect on root elongation and morphology [7,8]. The presence of an increased number of osteoclasts in alveolar bone (*Rank^Tg^* mice) was associated with earlier root elongation, while a reduction in this number (in *Rankl^+/-^* mice) was associated with delayed elongation [7,8,9]. Interestingly, it has also been demonstrated that the overactivation of the osteoclastogenic pathway stimulates the proliferation of the epithelial cells in Hertwig’s epithelial root sheath, in coordination with the mesenchymal cells in the apical papilla during the formation of the first third of the root [6,8]. This suggests that apical papilla tissue could be a regulatory center for root development and that the activity of the underlying alveolar bone could in turn control this regulation. An evaluation of the cellular response of the apical papilla in a context of osteoclastogenic inhibition would confirm this hypothesis.

While the roles of RANKL, RANK and OPG (osteoprotegerin, also named Tumor Necrosis Factor Receptor Superfamily Member 11B) in inflammation, immune response and bone resorption have been widely demonstrated, only a few studies have evaluated their direct involvement in dental morphogenesis and alveolar bone modeling [1,10,11,12,13,14]. During tooth morphogenesis and histogenesis, RANKL is expressed in the follicular mesenchymal cells and is co-expressed with OPG in the dental epithelium during the bud stage, while in the bell stage, both factors are expressed in the pulp cells, ameloblasts and odontoblasts [10]. RANK, for its part, is expressed in the epithelium of the tooth during the bud stage and later, during the bell stage, in the mesenchymal cells of the dental follicle. This co-expression illustrates the existence of local communication between the dental ectomesenchymal cells and alveolar bone cells. Recently, analyzing the craniofacial phenotype of second generation *Rankl^-/-^* mice validated the key role of RANKL/RANK signaling in dental morphogenesis [15]. Interestingly, blocking RANKL/RANK signaling in mice during the first postnatal week, by means of an anti-RANKL antibody, disrupts the normal eruption and root elongation of the first molar, demonstrating the crucial role of RANK signaling during this short specific time-window [16].

The objective of the present study was thus to assess the early stages of first molar root development in the osteopetrotic context of RANKL invalidation in order to demonstrate that the radicular phenotype observed is not only a result of defective osteoclasts, but also of the loss of cell-to-cell communications between dental, periodontium and alveolar bone cells, implicating RANKL/RANK signaling more particularly.

## 2. Results

### 2.1. Phenotypic Alterations in the Dento-Alveolar Bone Complex Associated with the Different Rankl Genotypes

**Figure 1:**
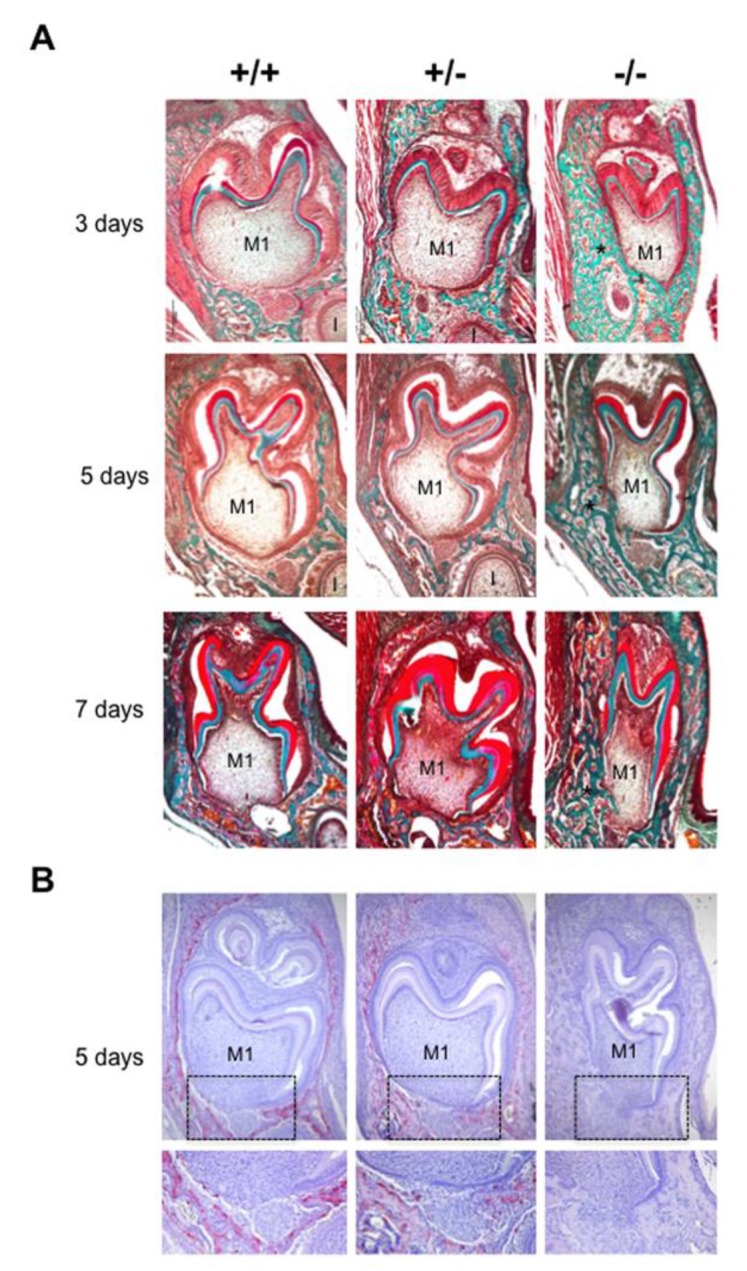
**Histological comparative analyses of the dental phenotype related to the different *Rankl* genotypes.** (**A**) Masson’s trichrome staining performed in 5µm thick frontal sections of the *Rankl*^+/+^, *Rankl*^+/-^ and *Rankl*^-/-^ C57BL/6 mouse heads at the ages of 3, 5 and 7 days postnatal. At least five mice were analyzed for each genotype and at each age. An equivalent distribution of the bone matrix around the first molars (M1) was observed for the *Rankl*^+/+^ and *Rankl*^+/-^ genotypes whatever the age considered. In contrast, for the *Rankl^-/-^* genotype, a grade increase in bone matrix deposition was observed with age (stars), so that the tissue boundary between the dental follicle and the apical papilla was interrupted, more specifically in the buccal region. Moreover, the *Rankl^-/-^* mouse molar presented over time progressive crown narrowing. I: incisor. Scale: 20X/100 µm. (**B**) TRAP histo-enzymology at postnatal day 5 evidenced a regular distribution of tartrate-resistant acid phosphatase staining (TRAP) positive osteoclasts around the first molar (M1) for the *Rankl*^+/+^ and *Rankl*^+/-^ genotypes. As expected, no TRAP staining was observed in the section of the *Rankl^-/-^* mice. A close view of the apical papilla (black rectangles) highlighted a reduction in TRAP staining in the *Rankl*^+/-^ section compared to the *Rankl*^+/+^ section. Scale 20X/100 µm.

The phenotype of mouse mandibular first molar and the underlying alveolar bone was histologically characterized for the three different *Rankl* genotypes (*Rankl^+/+^*, *Rankl^+/-^* and *Rankl^-/-^*) from postnatal days 3 to 7 (Figure 1A). Whatever age was considered, no histological difference was observed between the *Rankl^+/+^* and *Rankl^+/-^* mice (Figure 1A). In contrast, the *Rankl^-/-^* mice presented a severe osteopetrotic phenotype with alterations to both the crown and root tissue histogenesis, associated with grade entrapment in the surrounding hypertrophic alveolar bone (Figure 1A). Regarding the root, the formation of Hertwig’s epithelial root sheath (HERS) was initiated as visible at day 5 but was rapidly constrained by the presence of the hypertrophic alveolar bone, more obviously in the buccal region (Figure 1A). The tartrate-resistant acid phosphatase staining (TRAP) histo-enzymology was carried out on adjacent sections (presented for day 5 in Figure 1B), evidencing a total absence of staining in the *Rankl^-/-^* mouse regardless of age. Interestingly, while a similar distribution of TRAP positive cells was observed around the tooth in the alveolar bone of *Rankl^+/+^* and *Rankl^+/-^* mice, the number of positive cells, and the intensity of the staining, appeared lower in the heterozygous mice. This suggests the existence of a haplo-insufficiency effect, at least with regard to osteoclastogenesis (Figure 1B).

### 2.2. Alterations in Hertwig’s Epithelial Root Sheath Elongation Associated with the Different Rankl Genotypes

**Figure 2:**
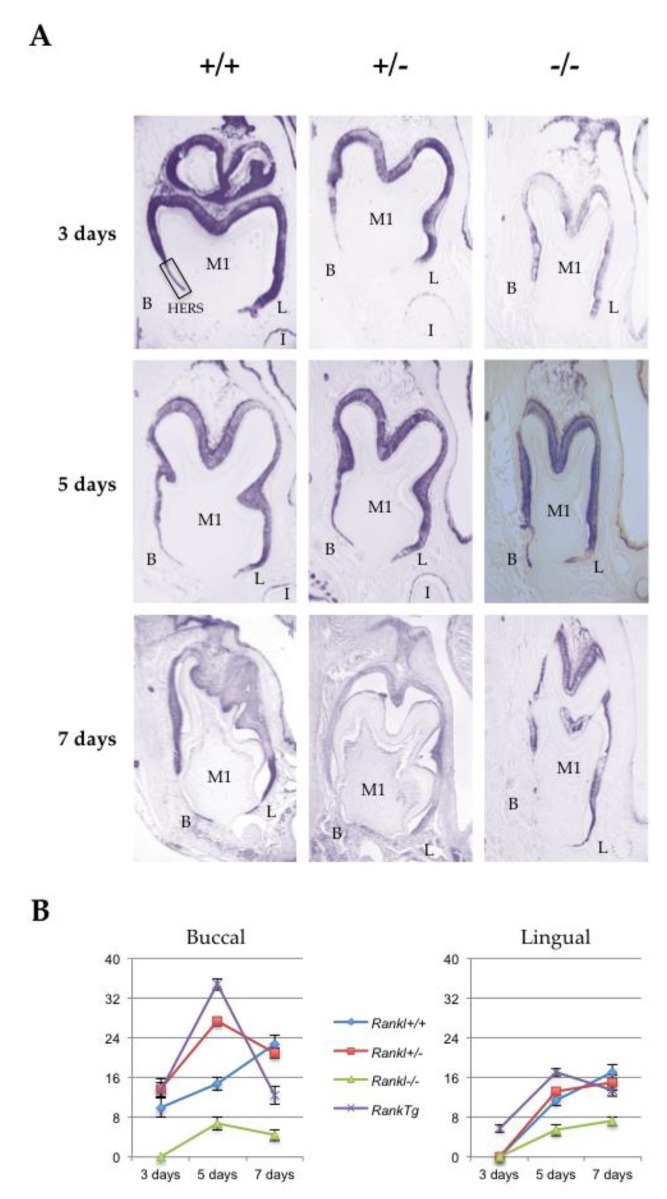
Comparative analyses of Hertwig’s epithelial root sheath length in the different *Rankl* genotypes based on keratin-14 immuno-staining. (**A**) Keratin-14 immunohistochemistry was performed on 5 µm thick frontal sections of the *Rankl*^+/+^, *Rankl*^+/-^ and *Rankl*^-/-^ C57BL/6 mouse heads at the ages of 3, 5 and 7 days postnatal. At least five mice were analyzed for each genotype and at each age. The *Rankl^-/-^* sections revealed a significant reduction in the number of Hertwig’s epithelial root sheath cells with interruptions in sheath continuity by bone matrix deposition with a progressive invasion of the pulp. In the lingual (*L*) region, the sheath appeared less altered morphologically than in the buccal (*B*) region. Scale: 20X/100 µm. (**B**) Graphic representation of the quantitative analyses of Hertwig’s epithelial root sheath cell numbers in the buccal and lingual regions performed on the sections of *Rankl*^+/+^, *Rankl*^+/-^ and *Rankl*^-/-^ C57BL/6 mouse heads at the ages of 3, 5 and 7 days postnatal. A similar quantitative analysis performed on a *Rank^Tg^* mouse with accelerated root elongation was added (five mice at each age). *Rankl^-/-^* sections presented a low number of epithelial cells, whether in the lingual or buccal regions, compared to either of the other two *Rankl* genotypes that appeared very similar, except on day 5 in the buccal region, or for the *Rank^Tg^* genotype.

Hertwig’s epithelial root sheath (HERS) elongation, which constitutes the main root developmental event, was characterized for the three different *Rankl* genotypes (*Rankl^+/+^*, *Rankl^+/-^* and *Rankl^-/-^*) from postnatal days 3 to 7 using the epithelial marker keratin-14 immunohistochemistry (Figure 2A). Measurements of the number of cells constituting the HERS (cells of the apical region of K14-positive epithelial organ (without ameloblasts) as presented in the photo of day 3 +/+ in Figure 2A) were made in the lingual and buccal regions of the mandible first molar mesial root for the different *Rankl* genotypes, as well as for the *Rank* overexpressing (*Rank^Tg^*) mouse that presented accelerated root elongation (Figure 2B and Table 1). Whatever region was considered, the number of cells was higher in the *Rank^Tg^* mice at days 3 and 5 compared to the control, while lower at day 7 indicating, as expected, earlier HERS elongation and disruption. In contrary, the number of cells was lower than in the control for the *Rankl^-/-^* mouse whatever day was considered, evidencing defective HERS elongation. Interestingly, the number of cells nevertheless grew continuously for the *Rankl^-/-^* mouse from days 3 to 7, at least in the lingual region, indicating a reduction rather than a blockage of HERS cell proliferation. Concerning the *Rankl^+/-^* mouse, a cell number similar to that of the control was observed, except at day 5 in the buccal region for an unexpected reason.

### 2.3. RANKL Expression Patterns and the Three Receptors, Namely RANK, OPG and LGR4 in the Mandible First Molar Mesial Root of 5-Day-Old Mice Supported the Implication of RANKL in the Proliferation of HERS Cells

**Figure 3:**
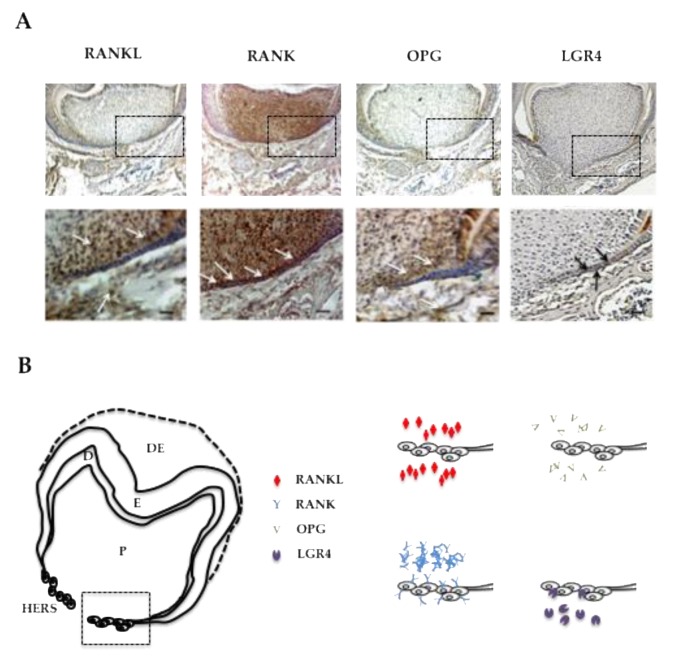
RANKL, RANK, OPG and LGR4 expression patterns in the mandible first molar of 5-day-old wild-type mice. (**A**) Immunohistochemistry experiments were carried out on 5-µm-thick frontal sections of 5-day-old wild-type C57BL/6 mouse heads for RANKL, RANK, OPG and LGR4. RANKL and OPG expressions were observed in some mesenchymal cells in the pulp, mainly facing Hertwig’s epithelial root sheath, in mesenchymal cells in the apical papilla, and in certain alveolar bone cells (arrowheads). RANK expression was high in the pulp, in Hertwig’s epithelial root sheath and in the cells at the bone surface. Mild LGR4 expression was evidenced in some cells in Hertwig’s epithelial root sheath and the apical papilla (arrowheads), and in most cells at the alveolar bone surface. Scale: 20X/100 µm. (**B**) Schematic representations of established RANKL, RANK, OPG and LGR4 expression patterns in 5-day-old wild-type C57BL/6 mice mandibular first molar roots. The cells in Hertwig’s epithelial root sheath expressed only RANK and LGR4, with a significant difference in the number of stained cells and the staining intensity in favor of RANK. DE: dental epithelium; E: enamel; D: dentin; P: pulp; HERS: Hertwig’s epithelial root sheath.

In order to characterize the potential direct implication of RANKL signaling in HERS elongation through modulation of the proliferation of epithelial cells from the apical area, the expression patterns of RANKL and its three known receptors RANK, OPG and LGR4 were established by immunohistochemistry on frontal sections of 5-day-old wild type mouse mandible first molars (Figure 3). RANKL expression was observed in the mesenchymal cells of the dental pulp facing the HERS, in cells from the apical follicular mesenchyme and in a few cells at the alveolar bone surface (Figure 3A). The main receptor RANK was highly expressed in the mesenchymal cells of the dental pulp, in the epithelial cells of HERS and in various large cells at the bone surface (Figure 3A). Interestingly, no RANK expression was detected in the cells in the apical follicular mesenchyme. Expression of the decoy receptor OPG was detected at low levels in the mesenchymal cells of the dental pulp facing HERS and in cells from the apical follicular mesenchyme (Figure 3A). Finally, expression of the alternative receptor LGR4 was detected at low levels in a few epithelial cells in HERS and at high levels in cells from the apical follicular mesenchyme and in cells at the alveolar bone surface (Figure 3A). The four expression patterns are summarized in Figure 3B for comparison. Regarding potential HERS cell proliferation modulation by RANKL signaling, it seems that control of this type implicates the RANK receptor, the one most expressed by those cells. Interestingly, concerning the two mesenchymal compartments, differential situations were observed. In the pulp compartment, RANK was the main receptor expressed whereas in the apical follicular mesenchyme, LGR4 was the main receptor expressed (Figure 3B), suggesting different impacts of RANKL on mesenchymal cells in those two compartments. Regarding the alveolar bone compartment, RANK was expressed in large cells at the bone surface, believed to be osteoclasts, while LGR4 was in rather small and numerous cells at the bone surface, suspected of being osteoblasts.

### 2.4. Alterations to HERS Cell Proliferation Associated with the Different Rankl Genotypes

**Figure 4:**
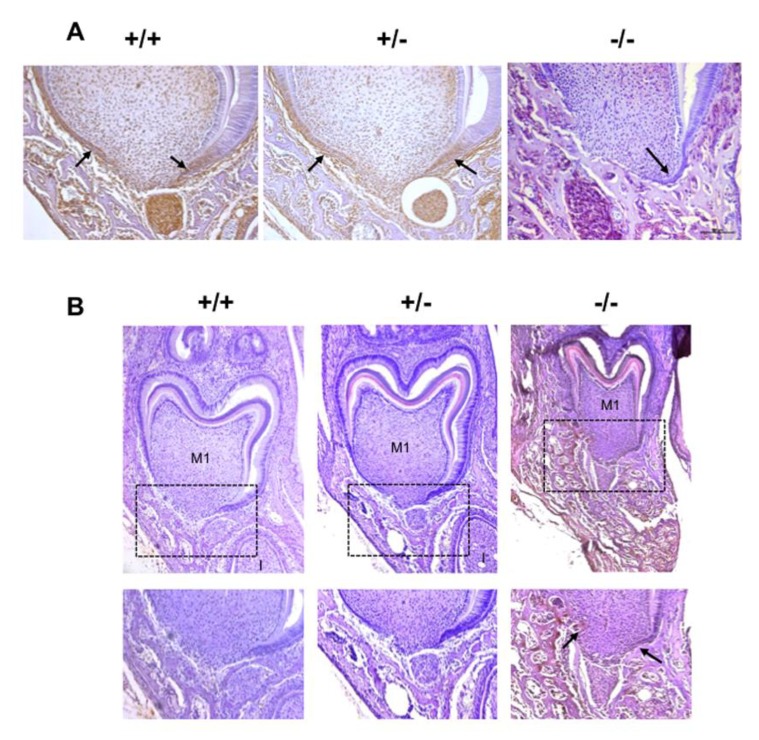
Proliferating cell nuclear antigen (PCNA) and P21 expression patterns in the mandible first molar of 5-day-old *Rankl*^+/+^, *Rankl*^+/-^ and *Rankl*^-/-^ C57BL/6 mice. Immunohistochemistry experiments were carried out on 5-µm-thick frontal sections of the *Rankl*^+/+^, *Rankl*^+/-^ and *Rankl*^-/-^ C57BL/6 mouse heads at the ages of 3, 4, 5, 6 and 7 days postnatal. At least five mice were analyzed for each genotype and at each age. Results obtained with 5-day-old C57BL/6 mice for PCNA (**A**) and P21 (**B**) were presented. PCNA expression was detected at similar levels for the *Rankl*^+/+^ and *Rankl*^+/-^ mice in the cells in Hertwig’s epithelial root sheath (arrows) and the cells in the apical papilla. In contrast, in the *Rankl*^-/-^ mice the PCNA staining was extremely reduced in those cells, but significant in the alveolar bone cells. P21 expression was almost absent in sections of *Rankl*^+/+^ and *Rankl*^+/-^ mice, but was significant for *Rankl*^-/-^ mice in the cells in Hertwig’s epithelial root sheath (arrows), the apical papilla and the alveolar bone. Black squares correspond to the region enlarged below. Scale 20X/100 µm. M1: first molar.

In order to characterize the potential modulation of HERS cell proliferation by RANKL signaling, the expression patterns of the proliferation marker proliferating cell nuclear antigen (PCNA) and the proliferation inhibitor P21^Waf-1/Cip-1/Sdi-1^ (P21) were established by immunohistochemistry on frontal sections of the mandible first molar mesial root of mice from the three different *Rankl* genotypes (*Rankl^+/+^*, *Rankl^+/-^* and *Rankl^-/-^*) at postnatal days 3 to 7. Histological results obtained at day 5 are presented in Figure 4 and the quantification/evaluation staining intensity in the HERS cells at all ages are presented in Table 2 and Table 3. Concerning PCNA expression, in the *Rankl^-/-^* mice, no expression was detected from day 5, whereas low staining was observed in a minority of mice at days 3 and 4 (Figure 4A and Table 2). 

In the *Rankl^+/+^* and *Rankl^+/-^* mice, PCNA staining was observed at all ages in HERS cells with a graded decrease in intensity from days 3 to day 7 (Figure 4A and Table 2). Interestingly, the intensity of the staining was globally lower in the *Rankl^+/-^* than in the *Rankl^+/+^* mice, in agreement with a haplo-insufficiency effect.

Concerning P21 expression, in the *Rankl^-/-^* mice, high intensity was observed in HERS cells from days 3 to 7 (Figure 4B and Table 3). In *Rankl^+/+^* mice, low intensity P21 expression was observed in HERS cells except at day 3, showing intermediary staining intensity (Figure 4B and Table 3). In *Rankl^+/-^* mice, low intensity P21 expression was observed in HERS cells except at day 3, for which P21 was not detected, and surprisingly at day 6, evidencing in most mice intermediary staining intensity (Figure 4B and Table 3).

### 2.5. Comparison of the Mandible first Molar Phenotypes between 35-Day-Old Wild Type, *Rankl^-/-^* and RANKL Transitory Defective Mice

**Figure 5:**
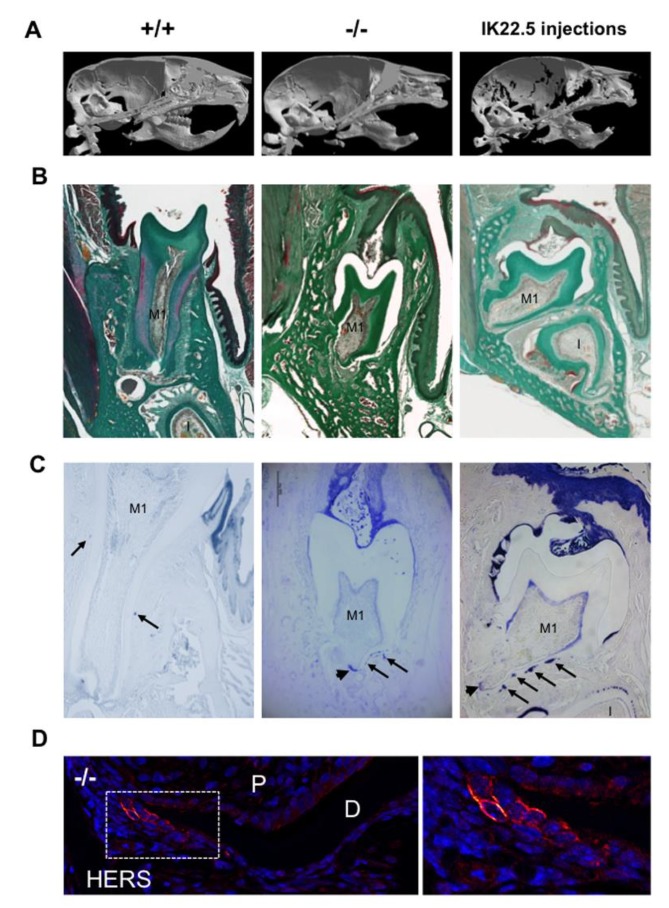
Comparative analyses in 35-day-old mice on the consequences on dental phenotype of the global (*Rankl^-/-^*) versus transient (4 injections of IK22.5 neutralizing antibody from postnatal days 1 to 7) invalidations of RANKL. At least five mice were analyzed for each group. (**A**) Micro-tomography 3D reconstructions of the heads of *Rankl*^+/+^, *Rankl*^-/-^ and RANKL transiently invalidated mice made it possible to observe similar phenotypes between the RANKL invalidated mice compared to the controls (*Rankl*^+/+^), with the presence of a more curved skull associated with dental eruption defects. (**B**) Masson’s trichrome staining carried out on frontal sections in the plane of the first molar (M1) of *Rankl*^+/+^, *Rankl*^-/-^ and RANKL transiently invalidated mouse heads confirmed the defective eruption and evidenced similar dysmorphic lower molars for the RANKL-invalidated mice in comparison to the controls. However, a more pronounced phenotype was visible for the global invalidation, with the presence of ankylosis in the buccal region. Interestingly, the incisor (I) was absent in this section’s plane only for the mouse globally invalidated for RANKL. Scale: 20X/100µm. (**C**) The keratin-14 immuno-labeling evidenced the presence of HERS cells and hypertrophic epithelial rests of Malassez cells (arrows), despite dysmorphic root formation and elongation in both RANKL-invalidated mice. Scale: 20X/100µm. (**D**) The keratin-14 immunofluorescence confirmed the presence of HERS cells in the most apical region of the root (apex) in the *Rankl^-/-^* mice at 35 days. D: dentin; P: pulp. The square corresponds to the enlarged region. Scale 20X/100 µm. M1: first molar; I: incisor.

In order to demonstrate that eruption and root elongation defects observed in the *Rankl^-/-^* mouse are due to defective expression/function of RANKL during the first postnatal week, the phenotypes of mice transiently invalidated for RANKL through IK22.5 RANKL neutralizing antibody injections during the first postnatal week were characterized and compared to *Rankl^-/-^* and WT mice at postnatal day 35. Compared to wild type mice, in permanently or transiently RANKL invalidated mice, dental eruption (Figure 5A–C) and root elongation (Figure 5B,C) were severely affected. For root elongation, the presence of HERS cells was observed in both types of invalidated mice (Figure 5C), more specifically in rests of Malassez (arrows in Figure 5C) and the most apical region of the sheath (arrowheads in Figure 5C,D) structures, suggesting incomplete root elongation, further supported by the absence of cellular cementum formation (Figure 5B). The number of HERS cells was greater in the transiently invalidated mice than in the permanently invalidated mice (Figure 5C), with an evident relationship to the length of the root that was greater in the transiently deficient mice (Figure 5B,C). Interestingly, other differences were observed between mice with global and transient RANKL invalidations. Ankylosis was only observed in the globally RANKL invalidated mice as a narrowed eruption pathway (Figure 5B). Altogether, these results revealed that RANKL transitory invalidation during the first postnatal week was sufficient on the one hand to block first molar eruption despite a large eruption pathway, and on the other to freeze root elongation in the absence of either ankylosis or compression inside hypertrophic osteopetrotic alveolar bone.

## 3. Discussion

In the present study, the early stages of first molar root development (first postnatal week) were compared between *Rankl* null mutant mice (*Rankl^-/-^*) and control wild type (*Rankl^+/+^*) and heterozygous (*Rankl^+/-^*) mice, in order to establish that the radicular phenotype observed was not only the result of defective osteoclasts responsible for the osteopetrotic phenotype, but also of the loss of certain cell-to-cell communications implicating the RANKL signaling. Expression patterns for the different receptors of RANKL were characterized at postnatal day 5 revealing three distinct situations. First, concerning the epithelial compartment, and more precisely HERS cells, the RANKL response was driven by the receptor that expressed the most, RANK. Secondly, concerning the mesenchymal compartment, the cells in the dental pulp appeared to be responsive to RANKL through the receptor RANK, whereas cells in the follicular sac were responsive through the receptor LGR4. Thirdly, in the bone compartment, osteoclasts and osteoblasts seemed to respond to RANKL through different receptors at this age, more precisely the osteoclasts through RANK and the osteoblasts through LGR4. Interestingly, concerning bone cells, RANK activation was crucial for osteoclast formation [17,18], whereas LGR4 activation promoted bone mass by stimulating bone mesenchymal stem cell differentiation into osteoblasts in response to R-spondin2, a competitor for RANKL for binding to LGR4 [19,20,21,22]. The alveolar bone phenotype observed in the absence of RANKL was coherent with the expression patterns of the two receptors in the bone cells. Our data show that defective stimulation of RANK induced an absence of osteoclasts and are in accordance with earlier prescriptive reported data [23]. The lack of RANKL might favor the binding of R-spondin2 to LGR4 at the osteoblast surface and consequently boosted bone formation.

Regarding the mesenchymal compartment, the complementary expression patterns of RANK and LGR4 in the dental pulp and dental follicle (observed at day 5 in the first molar) suggest the existence of a differential impact of RANKL on mesenchymal cells in both these sites with a probable histogenesis function. Interestingly, the expression pattern of LGR4, in the complex formed by the tooth, periodontium and alveolar bone, appeared to change sequentially from the initial morphogenesis stage [24] to the dental eruption stage [25,26], suggesting a dynamic/evolutive function of this receptor for RANKL and R-spondin2 throughout dental development, the modalities of which remain to be elucidated in future studies.

Concerning the epithelial compartment, RANK was the main receptor expressed in HERS cells on day 5. In *Rankl^-/-^* mice, a decrease in expression of the proliferation marker PCNA in the HERS cells was associated with an increase in expression of the cell-cycle progression inhibitor P21, explaining the important reduction in HERS cell proliferation. Interestingly, RANKL/RANK signaling has already been implicated in the control of epithelial cell proliferation in various organs, such as the mammary gland [27], the thymus [28] and the skin [29] suggesting that RANKL/RANK signaling may be crucial for controlling epithelial cell proliferation. The alterations to dental root development associated with functional RANKL/RANK signaling defects thus had two origins that could be classified as intrinsic to dental tissues, and extrinsic corresponding mainly to bone modeling/remodeling perturbations.

For the extrinsic origins, regardless of the way the bone remodeling is affected (e.g., a deficiency in RANKL/RANK inducing osteopetrosis [7,23,30,31,32,33] or overactivation in RANKL/RANK inducing osteolytic diseases [8,9]), in both cases root formation is touched. The most representative feature of a root developmental defect of extrinsic origin was the ankylosis observed in osteopetrosis.

For the intrinsic origins, which correspond to perturbations in the regulation of dental cell proliferation/differentiation by the RANKL/RANK/OPG/LGR4 system, the most representative feature of a root developmental defect was the slowdown of HERS elongation and ultimately of its disruption, with late maintenance of epithelial cells and defective cellular cementum formation. Interestingly, this relationship between HERS cell maintenance and the absence of cellular cementum was observed in mice continuously growing incisors, raising the question of the RANKL/RANK/OPG/LGR4 system’s potential implication in the continuously growing tooth. 

To conclude, the alterations in dental root development observed in *Rankl^-/-^* mice have two types of origin: intrinsic, corresponding to perturbations in dental-root-forming cell proliferation/differentiation, and extrinsic, corresponding to perturbations in these processes in other cells, more particularly bone cells (osteoclasts and osteoblasts). Vascular and immune cells will also need to be considered in the future, as vascularization and inflammation are important actors throughout development of the complex formed by the tooth, periodontium and alveolar bone.

## 4. Materials and Methods

### 4.1. Animals

The transgenic and control C57BL/6J mice were housed in pathogen-free conditions at the Experimental Therapy Unit at the Faculty of Medicine (Nantes, France) in accordance with the institutional guidelines of the French Ethical Committee (CEEA-PdL-06, accepted protocol number 01083.02 of 2014-01-14 then 18415-201901101823350v2 of 2019-06-09) and under the supervision of authorized investigators.

The *Rankl^-/-^* mouse line used in the study was initially generated by Y. Choi [23]. The genotyping was ensured by PCR using the following primers: 5′Rankl: CCAAGTAGTGGATTCTAAATCCTG; 3′Rankl: CCAACCTGTGGACTTACGATTAAAG; and 3′insert: ATTCGCAGCGCATCGCCTTCTATC as previously described [15]. At least five mice per group were analyzed.

Regarding transitory RANKL blockage with the IK22-5 antibody during the first postnatal week, the previously published protocol [16] was used, consisting of four subcutaneous injections at postnatal days 1, 3, 5 and 7, with 25 μg of IK22-5 antibody for the first and second injections (days 1 and 3) and 50 μg for the following (days 5 and 7). The mice were sacrificed 4 weeks after the last injection, at postnatal day 35. 

In all the experiments at least five mice were analyzed for each group (genotype or IK22-5 treatment) and at each age.

### 4.2. Micro-CT Analyses

Analyses of bone microarchitecture were performed using a Skyscan 1076 in vivo micro-CT scanner (Skyscan, Kontich, Belgium). Tests were performed after euthanizing the mice for each group. All heads were scanned using the same parameters (pixel size 9 µm, 50 kV, 0.5-mm Al filter, 20 min of scanning). The reconstruction was carried out using NRecon, and analyses were made using CTAn, CTVox and Dataviewer softwares (Skyscan).

### 4.3. Histology

The heads were collected from the euthanized mice and fixed in 4% buffered paraformaldehyde (PFA) in phosphate buffered saline 0.1M (PBS) for 48 hours. The heads were decalcified in 4.13% EDTA/0.2% PFA pH 7.4 in PBS for four days in a KOS sw10 (Milestone, Sorisole, Italy). The samples were dehydrated and embedded in paraffin or maintained in a PBS buffer solution at 4°C before cryostat sectioning. Then, 5µm-thick frontal sections stained with Masson’s trichrome were observed using a DMRXA microscope (Leica, Nussloch, Germany).

Tartrate-resistant acid phosphatase staining (TRAP, Sigma Chemical Co., St Louis, MO, USA) was performed as previously described [8] to identify the multinucleated osteoclast cells.

### 4.4. Immunohistochemistry

The paraffin was removed by immersion in toluene and the sections were rehydrated by immersing them in decreasing grades of ethanol solutions, followed by immersion in water and PBS 1X. When peroxidase-conjugated antibodies had to be used, a H_2_O_2_ (hydrogen peroxide) treatment was used for preliminary inactivation of the endogenous peroxidases. The sections were then saturated with serum (10%) and incubated with the primary antibody using the concentration indicated by the manufacturer (Table 4). One section in each slide was incubated without an antibody to serve as a negative control. After rinsing, sections were incubated with the secondary antibody at the dilution indicated by the manufacturer (Table 4). Horseradish peroxidase (HRP) enzymatic activity was determined using the NovaRED kit (Vector, Laboratories, Burlingame, USA) and the DAB kit (DAKO). The sections were then dehydrated and mounted with Eukitt (K, Freigburg, Germany). For the RANK, RANKL, OPG and LGR4 antibodies, the biotin-streptavidin peroxidase system was used as previously described [34].

In order to compare the number of epithelial cells present in Hertwig’s epithelial root sheath in the molars of *Rankl^-/-^* mice and the controls, a quantification of keratin-14 labeled cells was carried out for each molar and averaged according to the number of sections. The total numbers were compared between groups at all ages.

In order to compare the intensity of PCNA and P21 staining in the sections of the mandible of the *Rankl^-/-^* mice and the controls, the images were automatically digitized (Nanozoomer, Hamamatsu photonics) before quantification. Immunoreactivity was then analyzed qualitatively (cell type, location, nuclear/cytoplasmic staining) and semi-quantitatively. Semi-quantification was done for PCNA and P21 positivity using the following criteria: -, no HERS-cell stained; +, < 1/3 of the HERS-cells stained; ++, between 1/3 and 2/3 of the HERS-cells stained; +++, > 2/3 of the HERS-cells stained. For each case, the mean of the values in the five samples of each group was calculated. For observations and semi-quantifications, a double-blind examination by two researchers was carried out. 

### 4.5. Immunofluorescence

The decalcified mouse heads were immersed sequentially in 15% and 30% sucrose in PBS and mounted on Freeze Gel (Labonord, Z.I. de Templemars, France) for further cryostat sections. Sections were air dried and then saturated with 1% BSA in PBS for 30 minutes to block non-specific binding sites. Slides were incubated with a rabbit polyclonal primary antibody directed against keratin-14 (Covance AF64, Princeton, NJ, USA) diluted 1/500 in PBS at room temperature for 1 hour. After rinsing three times with PBS, the sections were incubated with a goat polyclonal anti-rabbit IgG secondary antibody coupled with Alexa Fluor 594 (A-11072, Life Technologies-ThermoFischer Scientific, Courtaboeuf, France) at room temperature for 1 hour and then rinsed and incubated for 10 minutes with DAPI (4,6-Diamidino-2-phenylindole dihydrochloride). After rinsing with PBS, the slides were mounted with cover slips and the fluorescence-mounting medium, Fluoprep (BioMérieux, Marcy l’Etoile, France). DAPI staining was used to evaluate cell density.

### 4.6. Statistical Analysis

The data obtained were analyzed using the IBM SPSS 20.0® program (SPSS. Inc., Chicago IL, USA). Descriptive statistics were used using means and standard deviations for quantitative variables (Hertwig epithelial sheath cell proliferation and positive TRAP cell numbers) and absolute frequencies for qualitative variables (expression pattern in apical papillae and HERS cells, intensities of P21 and PCNA protein labeling). In order to compare Hertwig epithelial sheath cell proliferation between different genotypes and their respective ages, an ANOVA test and Tukey’s multiple range (post hoc) test were used. A level of significance of 5% was always considered.

## Figures and Tables

**Table 1:** Quantification of epithelial cells in Hertwig’s epithelial root sheath (HERS) based on keratin-14 staining by genotype and age in both the lingual and buccal regions. Sections from five mice were analyzed for each genotype and at each age.

		*Rankl^+/+^*	*Rankl^+/-^*	*Rankl^-/-^*	*Rank^Tg^*
**LINGUAL**	3 days	0.0 ± 0.0	0.0 ± 0.0	0.0 ± 0.0	5.8 ± 0.8
	5 days	11.4 ± 1.1	13.2 ± 0.8	5.4 ± 1.1	17.2 ± 0.8
	7 days	17.2 ± 1.5	15.0 ± 1.6	7.2 ± 0.8	13.4 ± 1.1
**BUCCAL**	3 days	10.0 ± 2.0	13.8 ± 1.9	0.0 ± 0.0	13.6 ± 1.5
	5 days	14.8 ± 1.3	27.4 ± 0.9	6.8 ± 1.3	34.8 ± 1.1
	7 days	22.8 ± 1.9	21.0 ± 1.0	4.4 ± 1.1	12.4 ± 1.8

**Table 2:** Evaluation of PCNA labeling intensity in the apical area of the mandible first molar root according to genotype and age. Quantification was made on sections of five different mice for each group (genotype) and each age (all from postnatal days 3 to 7). -: no detection; +: low staining intensity; ++: intermediate staining intensity; +++: high staining intensity.

		PCNA Labeling Intensity			
		-	+	++	+++
*Rankl^+/+^*	3 days			1	4
	4 days		1	3	1
	5 days		2	2	1
	6 days		1	2	2
	7 days		3	2	
*Rankl^+/-^*	3 days		1	2	2
	4 days		2	1	2
	5 days		1	3	1
	6 days		2	2	1
	7 days		3	2	
*Rankl^-/-^*	3 days	3	2		
	4 days	4	1		
	5 days	5			
	6 days	5			
	7 days	5			

**Table 3:** Evaluation of P21 labeling intensity in the apical area of the mandible first molar root according to genotype and age. Quantification was made on sections of five different mice for each group (genotype) and each age (all from postnatal days 3 to 7). -: no detection; +: low staining intensity; ++: intermediate staining intensity; +++: high staining intensity.

		P21 Labeling Intensity			
		-	+	++	+++
*Rankl^+/+^*	3 days		1	4	
	4 days	1	4		
	5 days	1	4		
	6 days	1	4		
	7 days	1	3	1	
*Rankl^+/-^*	3 days	5			
	4 days	1	4		
	5 days	1	4		
	6 days	1		4	
	7 days	2	3		
*Rankl^-/-^*	3 days				5
	4 days				5
	5 days				5
	6 days				5
	7 days				5

**Table 4:** Primary and secondary antibodies and conditions of utilization.

Primary Antibodies			
**Anti-mouse RANK**	**Goat polyclonal IgG**	**R&D AF692**	1/20
Anti-human/mouse RANKL	Rabbit polyclonal IgG	Abcam ab62516	1/20
Anti-mouse OPG	Goat polyclonal IgG	R&D AF805	1/10
Anti-mouse LGR4	Rabbit polyclonal IgG	ThermoFischer PA5-67868	1/100
Anti-mouse PCNA	Rabbit polyclonal IgG	Abcam ab2426	1/500
Anti-mouse P21	Rabbit polyclonal IgG	Santa Cruz Biotechnology SC-397	1/50
Anti-mouse KERATIN-14	Rabbit polyclonal IgG	Covance AF64	1/500 - 1/1000
**Secondary antibodies**			
Bovine anti-goat	Biotin-SP conjugated polyclonal whole IgG	Jakson Immuno Research 805-065-180	1/400
Goat anti-rabbit	Peroxidase conjugated polyclonal IgG	Dako P0448	1/200 - 1/500
Horse anti-rabbit	Biotin conjugated polyclonal IgG	Vector laboratories BA-1100	7/1000
Goat anti-rabbit	Alexa Fluor 594 conjugated polyclonal IgG	Life Technologies A-11072	1/500

## Abbreviations

HERS	Hertwig’s epithelial root sheath
LGR4	Leucine rich repeat containing G protein-coupled receptor 4
OPG	Osteoprotegerin
PCNA	Proliferating cell nuclear antigen
P21	P21^Waf-1/Cip-1/Sdi-1^
RANK	Receptor activator of NF-κB
RANKL	Receptor activator of NF-κB ligand
